# Studying Effects of Calcium Oxide Nanoparticles on Dentinogenesis in Male Wistar Rats

**DOI:** 10.1155/2021/9983538

**Published:** 2021-07-24

**Authors:** Bushra Habeeb Al-Maula, Zena Jehad Wally, Mohanad Jameel Najm Al-Magsoosi, Rasha Hatem Dosh, Ruba M. Mustafa, Suhad Jabbar Hamed Al-Nasrawi, Abdullatif Alfutimie, Julfikar Haider

**Affiliations:** ^1^Scientific Center of Laser and Photonics, University of Al-Hamdaniya, Ninvah, Iraq; ^2^Department of Prosthodontic, Faculty of Dentistry, University of Kufa, Najaf, Iraq; ^3^Department of Oral Diagnosis, Faculty of Dentistry, University of Basrah, Basrah, Iraq; ^4^Department of Anatomy and Histology, Faculty of Medicine, University of Kufa, Najaf, Iraq; ^5^Department of Conservative Dentistry, Faculty of Dentistry, Jordan University of Science and Technology, Irbid, Jordan; ^6^Department of Conservative Dentistry, Faculty of Dentistry, University of Kufa, Kufa, Iraq; ^7^School of Chemical Engineering and Analytical Sciences, University of Manchester, UK; ^8^Department of Engineering, Manchester Metropolitan University, Manchester, UK

## Abstract

This study aimed to evaluate potential impacts of calcium oxide nanoparticles (CaO-NPs) at different dosages on predentin thickness, number of blood vessels, periodontal ligament thickness, and blood glucose level of Wistar rats. Twelve rats were randomly gathered into four groups, untreated (control) and CaO-NP-treated groups at three concentrations (25, 50, and 100 mg/kg of the body weight) over a period of 60 days. Histological investigation was performed on twenty-four lower incisor teeth extracted from all the tested groups under a light microscope, and an automatic Fujifilm was used to measure the blood glucose level. The results showed that regular nanoparticle treatment significantly increased predentin and periodontal ligament thicknesses, a gradual decrease in vascularization in the pulp tissue, and an increase in the blood glucose level as the dosages of nanoparticles administered to the rats increased. Administration of the CaO-NPs at low dosage (25 mg/kg) could be beneficial for the growth and integrity of teeth and dentinal tissues in rats.

## 1. Introduction

Calcium-based biomaterials such as calcium oxide (CaO) have been extensively used in dentistry and different biomedical applications due to their ability to release calcium ions after dissolution [1]. Calcium is the main component of the body's bones and teeth and plays an effective role in the functioning of different living cells such as osteoblast, ameloblast, and odontoblast, which form the calcified tissue of the bone, enamel, and dentin, respectively [2, 3]. Research was carried out to simulate the growth and mineralization of natural teeth with an aim of regenerating and repairing calcified tissues of the natural teeth [4].

Dentin is the second hardest tissue in the natural tooth after enamel and forms the overall bulk of the tooth and is not normally exposed to the oral environment [5]. The pattern and chemical composition of the dentin and bone matrix are somewhat similar. Dentin has a collagenous matrix rich in mineral crystal, which contains around 10% water, 20% organic materials, and 70% inorganic materials. The inorganic substance is made of calcium hydroxyapatite crystals, Ca_10_(PO_4_)_6_(OH)_2_, which are on and between the collagen fibers. These crystals are similar to those found in the enamel but smaller in size, making the dentin slightly softer than the enamel [2, 6].

Newly formed dentin called predentin forms at the expense of the dental pulp through a reparative, lifelong process called dentinogenesis [6]. With active dentinogenesis, the dentin layers calcify and mature throughout one's lifetime and maintain the teeth integrity [5]. Primary dentin is formed by odontoblasts which are estimated to form matrix molecules of approximately 10 *µ*m-thick layer and then deposited to about 4 *µ*m. In the root area, dentin was recognised by 15–30 *µ*m in thickness [2]. Dentin is formed by two main processes, collagenous matrix formation and mineral crystal formation in this matrix [7]. Dentin provides support to the superstructural enamel and shields the pulpal tissue from harmful microorganisms [8]. Reparative dentinogenesis is required to maintain pulp tissue vitality and protect it from the external environment [1]. In the root area, dentin is covered by the cementum where a group of connective tissue fibers called periodontal ligament (PDL) is bound and holds the tooth to the alveolar bone [9]. Mechanical stresses are transmitted and absorbed by the PDL which provides vascular supply and nutrients to the cementum and alveolar bone [10].

Calcium is a fundamental ion for dentinogenesis, and it is transported by the odontoblasts and blood serum-derived molecules under cellular control which normally maintains a balanced Ca concentration in the body. The extracellular accumulation of the calcium ions in the predentin led to the formation and maturation of the collagen scaffold network in the organic matrix of the dentin [7, 11]. Lacking in vitamin D or calcium intake may disturb the balance between the extracellular and intracellular calcium pools, and this may result in abnormal insulin release. Other studies also reported a correlation between vitamin D deficiency and impaired glucose-mediated insulin release [12].

Mineralized dentin is subjected to resorption by odontoblast cells with disturbed dentinogenesis. Deficiency in dentin formation may lead to pulp exposure, early loss of the overlying enamel, and subsequent tooth loss. Systemic conditions such as calcium and vitamin D deficiency and high blood glucose level may affect the process of dentin formation. Increase in the blood glucose level leads to bone destruction as in diabetes mellitus. Different vitamin D and calcium homeostasis showed a role in the development of type 2 diabetes mellitus [12]. Diabetes mellitus is considered as a risk factor for periodontitis in many research studies [13]. The periodontal ligament begins to replace bone loss by stretching and increasing PDL thickness. Periodontal ligament is a connective tissue structure which surrounds the root and is measured from the cementum to the bone surface of the socket.

Periodontal diseases are more common in people with diabetes. They occur due to changes in the function of immune cells, changes in blood vessels, and microflora in the gum and periodontium [14]. Periodontal diseases are painless, grow slowly, and cannot be observed in the early stages. However, in advanced stages, there will be bleeding, redness, and recession of the gingiva, bone destruction, teeth pockets and mobility, and eventually tooth loss [15].

Advances in nanotechnology are attracting researcher's interest due to the multifunctional properties of the materials in the nanoscale dimension. This permits altering some of the physical and chemical properties of the bulk materials such as surface area and structure. Calcium oxide nanoparticles (CaO-NPs) are inorganic and biocompatible materials showing an excellent antimicrobial activity against *Staphylococcus epidermidis*, *Pseudomonas aeruginosa*, and *Candida tropicalis* in addition to their ability to deactivate the endotoxins [16]. In a comparative study, CaO-NPs were found to be less effective than calcium hydroxide nanoparticles in eliminating bacteria in the dentinal tubules [17]. These particles were also found to be effective in decreasing the triglyceride and cholesterol amount of blood in mice [18]. Other studies reported that regular supplementation of calcium over a longer period may reduce the risk of fractures in healthy individuals [19]. Recent study stated that using saturated gum with 50% and 100% CaO-NPs might overcome the cariogenic challenge of the enamel in a dose-dependent manner [20]. However, the possible toxic mechanism of CaO-NPs is still a matter of concern, and further investigations are required in this area.

From the current literature, there is no clear evidence of the direct impact of the CaO-NPs on the dentinogenesis process and the associated structures. To the best of authors' knowledge, no other studies were found in the literature that investigated the effects of oral administration of CaO-NPs on the tooth structure in order to determine the optimal dosage. Thus, the present study aimed to evaluate the impact of a regular administration of the synthesised CaO-NPs at different dosages (25, 50, and 100 mg/kg) on the predentin thickness, periodontal ligament thickness, number of blood vessels, and blood glucose level of Wistar rats over a period of 60 days.

## 2. Materials and Methods

### 2.1. Preparation of Calcium Oxide Nanoparticles

To prepare the experimental nanomaterials (CaO-NPs), 1.5 g of CaCl_2_.2H_2_O (BDH Chemicals Ltd., Poole, England) powder was dissolved in 50 mL of redistilled water. The resulting solution was transferred to a round flask with stirring, and 15 mL of reduction agent, NaOH (1M), was rapidly added to the mixture to form a nanoparticle suspension. This suspension was kept in a water bath for an hour at 75°C and then allowed to cool to room temperature. Using a centrifuge, the nanoparticles were separated. To remove any contamination, the particles were washed with doubled distilled water and then dried in an oven at 80°C. A desired amount (7.5 mg) of nanoparticles was mixed with 30 ml of redistilled water with sonication for 5 hours to eliminate the agglomeration and make the colloid containing individual nanoparticles.

Using an X-ray diffractometer, a spectrophotometer, and an atomic force microscope, CaO-NPs have been characterized and confirmed in a previous work [21] at the Faculty of Dentistry, University of Kufa, Iraq. The nanoparticles were distributed in a size ranging between 15 nm and 65 nm.

### 2.2. Experimental Design and Procedure

**Figure 1:**
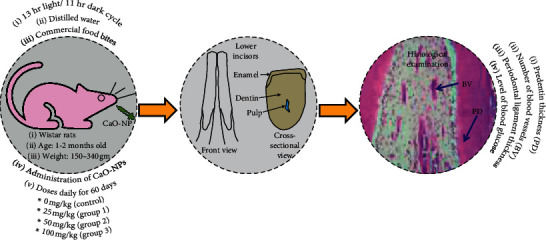
Experimental design to assess the effects of CaO nanoparticle (NP) administration on dentinogenesis of Wistar rats.

Experimental procedures were performed in this research by following the legislation of animals used for scientific research (Directive 2010/63/EU of the European Parliament and of the Council of 22 September 2010) with ethical approval granted by the University of Kufa (reference no. 3202). In the present study, 12 male Wistar albino rats aged between 1 and 2 months with a mass of 150–340 gm originating from the University of Kufa, Iraq, were considered. During the experimental period, the rats were housed in a plastic cage at the University of Kufa, Iraq, under standard laboratory conditions of 13 hr light and 11 hr dark cycles. Distilled water and commercial food bits were used to feed the rats. Then, the animals were randomly divided into 4 groups, each containing 3 rats. Experimental groups were treated with the CaO-NP suspension at different dosages (25, 50, and 100 mg/kg of the body weight). Using oral intubation, the rats were administrated CaO-NPs daily for 60 days. Normal saline (0.9%) was used to treat the control group using the same timing protocol. At the end of the study, all the rats were scarified using intramuscular anaesthesia (0.25 ml zylazine/100 g and 5 mg ketamine/100 g of the body weight) to test the influence of CaO-NP administration on the predentin thickness, number of blood vessels in the pulp, periodontal ligament thickness, and level of blood glucose. The experimental design is presented in Figure 1.

#### 2.2.1. Histological Examination

The histological examination was carried out on 24 lower incisors of all the evaluated groups. Two aspects of each tooth were tested; thus, in total, 48 tissue samples were examined. The sample size was decided after running a pilot study on a pair of teeth from each group, where the standard deviations and the mean differences were (5.66, 28.5), (2.12, 4), and (0.71, 3.5) for predentin thickness, periodontal ligament thickness, and blood glucose, respectively. The two lower incisors with jaw bones were carefully removed from each scarified animal with disposable knives (Sigma, Germany), and the soft tissue was striped to expose the root part of the teeth and surrounding bone. Hard tissue specimens were immediately fixed and stored at 10% freshly prepared formalin for 3 days. For decalcification, the specimens were left in a formic acid-sodium citrate solution, which was freshly prepared from two different solutions (125 cc 90% formic acid and 125 cc distilled water and (50 mg sodium citrate and 250 cc distilled water). The decalcification solution was changed after every 3-4 days, and the specimens were checked periodically using a fine needle. Decalcification was achieved when the needle was able to deeply penetrate the specimen without any resistance. Then, the specimens were placed under running water for half an hour to remove the residual acid [22].

To prepare the experimental specimens for histological examination, initially, they were subjected to a gradual dehydration over a series of alcohol-water solutions (60%, 80%, 90%, and 100%) for 2 hours. Alcohol in the specimens was then replaced by 2 changes of xylene which was readily soluble in alcohol. After that, the specimens were placed in a dish of fresh paraffin wax, and the dish was transferred to an oven at a constant temperature of 50-53°C. The specimens were then transformed to 2 or 3 successive dishes of paraffin to remove xylene in the tissue and be replaced by paraffin. Later, the specimens were embedded in the center of the paraffin block and adjusted to a microtome (Leitz/Germany) where serial sectioning of 5 *μ*m thickness for each part of the bone containing the tooth was done. Hematoxylin and eosin stains were finally used to stain the specimens which were then mounted on clean glass slides (Sail Brand/China) for histological examination using a light microscope.

**Figure 2:**
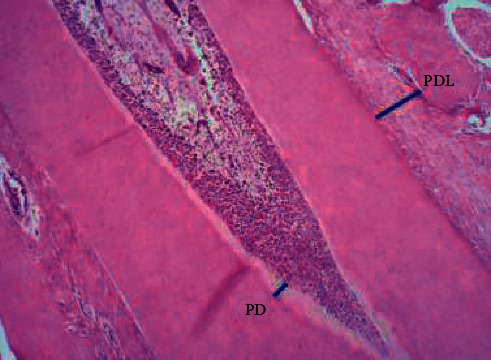
Analysis of the histological images by ImageJ software measuring thickness of the predentin (PD) and periodontal ligament (PDL).

Histomorphometric assessment for the thickness of predentin and periodontal ligament was performed with a micrometer scale by ImageJ software (NIH, USA) as shown in Figure 2, whereas the number of blood vessels in the pulp area was histologically counted by taking two fields of each slide using a light microscope with a magnification of ×10.

#### 2.2.2. Measuring the Blood Glucose Level

The samples of blood were collected from the hearts of both healthy control and CaO nanoparticle-treated groups via cardiac puncture using 23 G 1¼ʺ needles. The level of blood glucose was measured by an automatic Fujifilm (DRI-CHEM NX500), Japan.

### 2.3. Statistical Analysis

The collected data were displayed as mean ± standard deviation (SD). The statistical analyses of the experimental values were compared to the control. Data were analyzed by GraphPad Prism 8.4.3 software using one-way ANOVA with Tukey's multiple comparison post hoc test. *P* < 0.05 was determined to identify statistically significant differences.

## 3. Results

### 3.1. Effect of CaO-NPs on Predentin Thickness and Dental Pulp Vascularization

**Figure 3:**
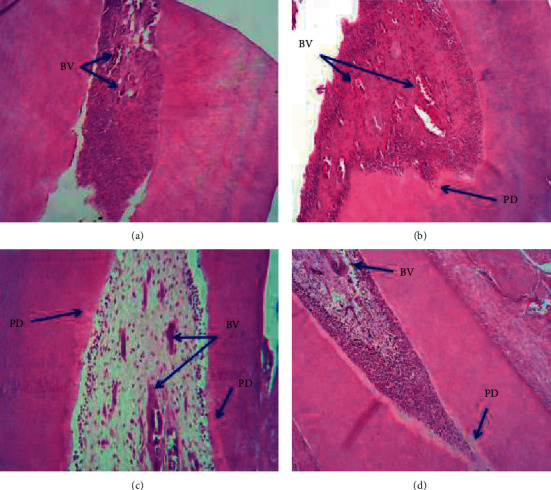
Representative histological images under the light microscope (H&E, ×10) showing (a) dental pulp at the control group with very thin layers of the predentin and a high number of blood vessels, (b) 25 mg/kg CaO-NP treatment with a slightly thicker predentin and a low number of blood vessels, (c) 50 mg/kg CaO-NP treatment with a slightly thicker predentin and a low number of blood vessels, and (d) 100 mg/kg CaO-NP treatment with a thick and irregular predentin (PD) and very low number of blood vessels (BV).

Histological examination images of the animal tissues in the pulpal region after 60 days of daily administration of CaO-NPs at various dosages (25, 50, and 100 mg/kg) and the nontreated (control) group are presented in Figure 3. The histological images revealed a general increase in the predentin formation activity and a progressive decrease in the vascularization with the increase in the concentration of CaO-NP administration.

**Figure 4:**
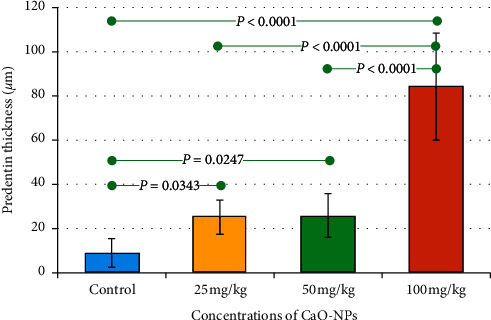
Bar chart showing mean values of predentin thickness in the teeth of Wistar rats after 60 days of CaO-NP administration at different dosages (25, 50, and 100 mg/kg) and without any nanoparticle administration (control group). Horizontal lines connecting two bars indicate statistical significance (*P* < 0.05).

Interestingly, the maximum predentin thickness (84.25 ± 24.20 *μ*m) was reported in animals treated with 100 mg/kg CaO-NPs, while the least thickness (9.00 ± 6.50 *μ*m) was found in the nontreated control group as shown in Figure 4 and Table 1. There was a statistically significant difference in predentin thickness between the control group and all CaO-NP-treated groups at varying dosages (25, 50, and 100 mg/kg).

**Figure 5:**
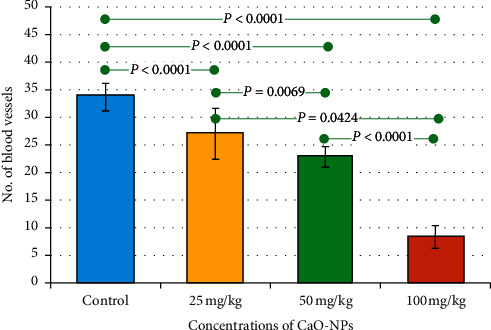
Bar chart showing mean values of the number of blood vessels in the dental pulp of Wistar rats after 60 days of CaO-NP administration at different dosages (25, 50, and 100 mg/kg) and the nontreated control group. Horizontal lines connecting two bars indicate statistical significance (*P* < 0.05).

Analysis of the histological images revealed a progressive decrease in the vascularization with the increasing dosages of CaO-NPs. The highest number of blood vessels (33.67 ± 2.49) was counted in the nontreated control group. This number declined gradually in the rats treated with CaO-NPs in a dose-dependent manner to reach the lowest number (8.3 ± 2.06) at 100 mg/kg CaO-NP administration as shown in Figure 5 and Table 2. There was a statistically significant difference in the number of blood vessels between CaO-NP-treated groups at variable dosages (25, 50, and 100 mg/kg) and the control group.

### 3.2. Effect of CaO-NPs on Periodontal Ligament (PDL) Thickness

**Figure 6:**
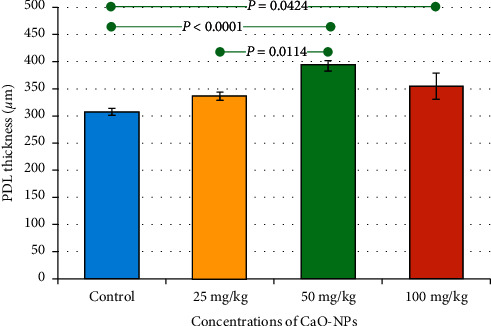
Bar chart showing mean values of the periodontal ligament (PDL) thickness in the teeth of Wistar rats after 60 days of CaO-NP administration at different dosages (25, 50, and 100 mg/kg) and the nontreated control group. Horizontal lines connecting two bars indicate statistical significance (*P* < 0.05).

Histological investigation of the pulp tissue in the tested rats showed an overall increase in the thickness of the periodontal ligament (PDL) with the increase in CaO-NP concentration as presented in the bar chart in Figure 6.

Rats treated with 50 mg/kg CaO-NPs reported the highest periodontal thickness (392.3 ± 34.80 *μ*m), whereas the minimum thickness (307.6 ± 58.62 *μ*m) was found in the nontreated control group as shown in Table 3. There was a statistically significant difference in periodontal ligament thickness between the control group and the groups treated with variable dosages of CaO-NPs (50 and 100 mg/kg).

### 3.3. Effect of CaO-NPs on the Level of Blood Glucose

**Figure 7:**
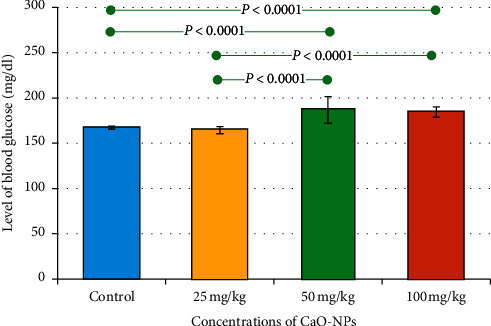
Bar chart showing mean values of the blood glucose level in the teeth of Wistar rats after 60 days of CaO-NP administration at different dosages (25, 50, and 100 mg/kg) and the nontreated control group. Horizontal lines connecting two bars indicate statistical significance (*P* < 0.05).

The influence of CaO-NP administration on the level of blood glucose of the tested rats is presented in Figure 7 and Table 4. Interestingly, the blood glucose level increased gradually with the increase in dosages of CaO-NPs. As can be seen in Table 4, the nontreated group reported the lowest level (167.3 ± 1.63 mg/dl), while the group treated with 50 mg/kg CaO-NPs showed the highest glucose value (186.8 ± 16.19 mg/dl). Figure 7 shows statistically significant differences between the untreated control group and most of the other tested groups.

## 4. Discussion

CaO-NPs have been used for various biomedical applications such as drug delivery and antimicrobial agent [23]. In the literature, different animal studies found that the administration of CaO-NPs had a significant impact on the functioning of different body parts such as the liver, blood cholesterol, and body weight [18]. Using saturated gum with CaO-NPs was also found to be effective to induce remineralization of initial caries of extracted human teeth [20]. Furthermore, it was stated that nanotechnology facilitates oral administration of different drugs [24, 25]. Other nanoparticles containing poly(d,l-lactide-co-glycolide) acid and lovastatin were used as a direct pulp capping and found effective to induce odontoblastic differentiation, dentinogenesis, and angiogenesis at the pulp exposure site in rat teeth [26]. However, cytotoxicity of statin at high concentrations has been a matter of concern in some studies [26, 27]. Therefore, the current study was aimed to evaluate the influence of daily administration of CaO-NPs at different dosages (25, 50, and 100 mg/kg) on predentin thickness, periodontal ligament thickness, number of blood vessels, and blood glucose level of Wistar rats over a period of 60 days. CaO-NPs were chosen here due to the presence of the calcium element with the potential of its cellular uptake, thus improving dentinogenesis.

The results of this study revealed an overall increase in the predentin formation activity and periodontal ligament thickness as the concentration of CaO-NPs administered to the Wistar rat increased, whereas vascularization in the pulp area showed a gradual decrease in a dose-dependent manner. Blood glucose reported high levels with the increase in CaO-NP dosages.

Histological examination of the current research showed that daily treatment of CaO-NPs at 100 mg/kg in Wistar rats over a period of 60 days produced the highest predentin thickness (84.25 ± 24.20 *μ*m) compared to the untreated (control) group (9.00 ± 6.50 *μ*m). The long-term treatment of the rats with CaO-NPs might increase the level of calcium. Lower calcium level has shown to inhibit proper tooth mineralization [28]. Calcium ion circulates by cellular uptake through the dentin-forming cells, odontoblasts, which transport calcium ions towards the mineral formation site in the dentin matrix [11, 29]. In another study, the release of Ca and OH ions from the pulp capping materials was capable to encourage the deposition of hard tissue [30]. Ca ions were also shown to reduce the capillary permeability which diminished the serum flow and inhabit the levels of inhibitory pyrophosphates which caused the mineralization [31].

The progressive decrease in the number of blood vessels by 8.3 ± 2.06 at 100 mg/kg CaO-NP treatment compared to the highest value (33.67 ± 2.49) recorded for the control group could be attributed to the continued deposition of predentin which was shown in the results. Significant reduction in pulp vascularization was observed with thicker dentin layers.

Periodontal ligaments occupy the distance between the bone and cementum and hold the root of the tooth to the underlying bone [9]. In the present study, this distance was found thicker when the rats treated with CaO-NPs over 60 days were compared to the untreated group. The rats treated with 50 mg/kg CaO-NPs reported the highest (392.3 ± 34.80 *μ*m) PDL thickness. This could be in response to the bone destruction at the root area where the PDL stretches to substitute the bone loss. The administration of CaO-NPs was also found significant in increasing the level of blood glucose by 186.8 ± 16.19 mg/dl in comparison to the untreated group (167.3 ± 1.63 mg/dl). Insulin secretion is a calcium-dependent process [32]. The rapid increase in intracellular calcium ions activates the insulin release [33]. High blood glucose level has been associated to bone destruction in diabetes mellitus [12].

In the current study, the regular administration of CaO-NPs showed an effective elevation of predentin formation activity and periodontal ligament thickness. The optimum concentration of CaO-NPs was found with the dosage of 25 mg/kg as it caused a significant increase in the predentin thickness with the least decrease in vascularization and no significant influence on the PDL thickness and the blood glucose level in comparison to the nontreated group. However, one limitation was the duration of the experiment considered in this study. In future, further investigations could be carried out to study the effect of CaO-NPs for a shorter period on the predentin and to find out the effect on other associated tooth structures, such as the cementum, surrounded bone tissue, and oral mucosa, and possible cytotoxic effect.

Despite the wide use of CaO-NPs, their mechanisms of action remain unclear. Further studies are required to broaden the understanding of mechanisms associated with induced dentinogenesis. This will help in optimizing the currently available biomaterials to target specific regenerative processes for obtaining the best possible clinical outcomes.

Oral administration of CaO-NPs with controlled dosage might have a role in dentinogenesis, enhancing dentin mineralization and counteracting the cases of dentin resorption of the human teeth.

## 5. Conclusion

The influence of daily administration of CaO-NPs at different concentrations (25, 50, and 100 mg/kg) on predentin thickness, number of blood vessels, periodontal ligament thickness, and blood glucose level of Wistar rats over a period of 60 days was evaluated compared to the untreated rats. The results showed a general increase in predentin formation activity and periodontal ligament thickness as the concentration of CaO-NPs administered to the rats was increased. Although vascularization in the pulp area showed a gradual decrease in a dose-dependent manner, high levels of blood glucose were reported with the increase in CaO-NP dosages. The optimum dosage of CaO-NPs was reported at the lowest concentration (25 mg/kg) for better dentinogenesis. Therefore, treatment with such biocompatible and inexpensive nanomaterials could be promising for improving the growth and integrity of the tooth and dentin tissue.

## Figures and Tables

**Table 1:** Mean values of predentin thickness in the teeth of Wistar rats after 60 days of CaO-NP administration at different dosages (25, 50, and 100 mg/kg) and without any nanoparticle administration (control group).

Groups	Control (0 mg/kg)	25 mg/kg	50 mg/kg	100 mg/kg
Number of thickness data	12	12	12	12
Mean thickness (*µ*m)	9.00^a^	25.92^b^	25.92^b^	84.25^c^
Std. deviation (*µ*m)	6.50	7.73	9.84	24.20
Std. error (*µ*m)	1.879	2.232	2.843	6.987
Minimum (*µ*m)	0.0	16.00	12.00	48.00
Maximum (*µ*m)	18.0	38.00	51.00	121.00

^a–c^Different letters indicate statistical significant differences (*P* < 0.05).

**Table 2:** Mean values of the number of blood vessels in the dental pulp of Wistar rats after 60 days of CaO-NP administration at different dosages (25, 50, and 100 mg/kg) and the nontreated control group.

Groups	Control	25 mg/kg	50 mg/kg	100 mg/kg
Number of blood vessels' data	12	12	12	12
Mean	33.67^a^	27.0^b^	22.8^c^	8.3^d^
Std. deviation	2.49	4.63	1.85	2.06
Std. error	0.7213	1.337	0.5342	0.5946
Minimum	31.0	17.0	20.0	5.0
Maximum	38.0	31.0	25.0	11.0

^a–d^Different letters indicate statistical significant differences (*P* < 0.05).

**Table 3:** Mean values of PDL thickness in the teeth of Wistar rats after 60 days of CaO-NP administration at different dosages (25, 50, and 100 mg/kg) and the nontreated control group.

Groups	Control	25 mg/kg	50 mg/kg	100 mg/kg
Number of thickness data	12	12	12	12
Mean thickness (*µ*m)	307.6^a^	336.3^a,c^	392.3^b^	354.8^b,c^
Std. deviation (*µ*m)	58.62	32.71	43.80	26.78
Std. error (*µ*m)	16.92	9.444	12.64	7.732
Minimum (*µ*m)	195.0	292.0	313.0	324.0
Maximum (*µ*m)	409.0	374.0	440.0	414.0

^a–c^Different letters indicate statistical significant differences (*P* < 0.05).

**Table 4:** Mean values of the blood glucose level in Wistar rats after 60 days of CaO-NP administration at different dosages (25, 50, and 100 mg/kg) and the nontreated control group.

Groups	Control	25 mg/kg	50 mg/kg	100 mg/kg
Number of blood glucose data	12	12	12	12
Mean glucose (mg/dl)	167.3^a^	164.5^a^	186.8^b,d^	184.3^c,d^
Std. deviation (mg/dl)	1.67	3.77	14.76	5.75
Std. error (mg/dl)	0.4820	1.091	4.262	1.662
Minimum (mg/dl)	164.0	160.0	173.0	175.0
Maximum (mg/dl)	169.0	169.0	214.0	190.0

^a–d^Different letters indicate statistical significant differences (*P* < 0.05).

## Data Availability

The data that support the findings of this study are available from the corresponding author upon reasonable request.
